# Making implementation science more real

**DOI:** 10.1186/s12874-022-01661-2

**Published:** 2022-06-25

**Authors:** Mitchell N. Sarkies, Emilie Francis-Auton, Janet C. Long, Chiara Pomare, Rebecca Hardwick, Jeffrey Braithwaite

**Affiliations:** 1 Centre for Healthcare Resilience and Implementation Science, Australian Institute of Health Innovation, Faculty of Medicine, Health and Human Sciences, Macquarie University, 75 Talavera Road, Sydney, New South Wales 2109 Australia; 2 Peninsula Medical School, Faculty of Health, University of Plymouth, Plymouth, UK

**Keywords:** Implementation science, Realist evaluation, Realist review, Realist synthesis, Context, Mechanism, Outcome, Theory

## Abstract

Implementation science in healthcare aims to understand how to get evidence into practice. Once this is achieved in one setting, it becomes increasingly difficult to replicate elsewhere. The problem is often attributed to differences in context that influence how and whether implementation strategies work. We argue that realist research paradigms provide a useful framework to express the effect of contextual factors within implementation strategy causal processes. Realist studies are theory-driven evaluations that focus on understanding *how* and *why* interventions work under different circumstances. They consider the interaction between contextual circumstances, theoretical mechanisms of change and the outcomes they produce, to arrive at explanations of conditional causality (i.e., what tends to work, for whom, under what circumstances). This Commentary provides example applications using preliminary findings from a large realist implementation study of system-wide value-based healthcare initiatives in New South Wales, Australia. If applied judiciously, realist implementation studies may represent a sound approach to help optimise delivery of the right care in the right setting and at the right time.

## Contributions to the literature

- Replicating implementation successes from one healthcare setting to another remains an ongoing challenge for implementation science.
- Existing efforts to understand the process of implementation, and thereby facilitate the generalisation of implementation strategy success, has relied on dividing and studying contextual factors, theoretical mechanisms, and outcomes in isolation from one another.
- Realist research paradigms offer an analytical tool to articulate the explicit role of context within the causal process, moving beyond a catalogue of preconditions for implementation success.

## Background

### There are still no magic bullets

One consistent insight from implementation science in healthcare is that widespread practice change is difficult to achieve [1]. Small scale, bottom-up improvements, while successful in one site, might struggle to achieve the same benefits when trialled elsewhere [2]. Even in the presence of evidence consensus, system-wide transformations in care are hampered by competing policy priorities, entrenched cultural norms, and mismatched resources, amongst other factors. These challenges create a wedge between system-wide efforts to improve care that don’t always match local needs and on-the-ground solutions that might not be readily scalable.

Faced with seemingly insurmountable challenges, it can be tempting to treat the problem as an operationalisation issue that can be solved with a blueprint of steps to follow or prescriptive checklists of successful ingredients. Over-reliance on these cookie-cutter types of approaches potentially conceals the deeply complex social process of implementation [3].

We can observe this in the deployment of strategies to support improvement initiatives which do not necessarily match the barriers faced by clinicians at the point of care. In healthcare settings, implementation strategies comprise the specific means or methods for adopting and sustaining evidence-based programs or interventions [4]. It is not uncommon for education or audit and feedback implementation strategies (which work through individual behaviour change) to be applied indiscriminately to address issues at the organisational level where people might be bedevilled by a lack of local infrastructure or policy that is not-fit-for-purpose [5]. Many clinicians will be familiar with the hand hygiene auditor hovering over their shoulder, which plays an important role in reinforcing good hand hygiene practice; but it does not address whether the hand sanitiser dispenser is conveniently located and regularly refilled—or there at all. What this illustrates is that not all improvement initiatives are built the same; they produce disparate outcomes in different circumstances [6]. To replicate the more successful approaches at scale, we need to shift our thinking from the *composition* of improvement initiatives to their *properties*. In other words, rather than seeking to understand every contextual element at play during implementation it would be more informative to discern the specific influence of contextual differences on the causal processes triggered by implementation strategies. For example, unpicking *how* implementation strategies like audit and feedback produce favourable outcomes [7–9], as well as *why* they work in different ways depending on the contextual circumstances at play.

We argue that the key to producing generalisable knowledge and predicting how large-system change might unfold lies in answering both these *how* and *why* questions together. These questions are inextricably linked; neither operates independently like they do in relation to one another. Get this right and we can craft context-specific packages of implementation strategies, which squarely address variation in implementation outcomes across sites.

Realist research paradigms [10] are permeating the implementation science literature. However, they more often focus on the examining a particular healthcare program, and are rarely applied to understanding the strategies used to implement those programs. Realist research, which is grounded in the philosophy of scientific realism [11], offer a nuanced understanding of *how* and *why* different implementation science programs work under varying circumstances [12]. Realist studies are a form of theory-driven research that assume interventions only work under certain circumstances and that their success is influenced by the way different agents respond to them [13]. They assert that interventions always have a theoretical underpinning, whether explicit or implicit, and seek to test this theory against empirical evaluation of how the intervention works and what conditions may hinder or promote successful outcomes [14, 15].

Realist research can offer a framework to understanding *how* and *why* implementation strategies work by linking three core pillars of evaluation [16]: 1) contextual circumstances that are conducive to strategies triggering change, 2) strategy mechanisms causing those change processes, and 3) outcomes produced by interactions between those processes in conducive contextual circumstances [17]. Realist studies have been used to evaluate programs, interventions and policies across a range of fields (public health, health services research, criminal justice and international development, to name a few). Applying the same concepts to understanding implementation strategy mechanisms may bear fruitful gains for improving healthcare systems. The purpose of this Commentary is to step back, take stock, and articulate precisely what realist studies have to offer this field.

### Context dependency

Within implementation science, contextual factors, theoretical mechanisms, and implementation outcomes achieved through different implementation strategies are usually divided and studied in isolation [18]. We argue that these elements cannot be decoupled from one another and should be examined in relation to each other. The empirical knowledge base supporting methods for acquiring and applying evidence in healthcare has seen rapid growth, through groups such as Cochrane’s Effective Practice and Organisation of Care. However, effect estimates from implementation strategies are rarely partnered with rich explanations for how these outcomes were achieved [19]. Establishing a causal association between implementation efforts and observed outcomes in situ is not universal; they are almost always contingent on the social processes generating changes and the environmental circumstances necessary to trigger those processes. For example, *Matching Michigan* [20], a two-year concerted intervention in 223 intensive care units (ICUs) in England to reduce bloodstream infections from central venous catheters, reported a generalised, “rising tide”, secular trend in improvement. But in *Explaining Matching Michigan* [21], one of the enrolled ICUs achieved its outcomes by changing practices and culture in line with the intervention; five strengthened existing practices and 11 did not respond to the package of interventional initiatives.

Context is often examined separately as compiled lists of important factors, which are not explicitly linked to *why* these may differ between sites and *how* they influence the social processes that impact outcomes [22]. Understanding the conditions for implementation success have enabled the creation of conducive environments for change [23, 24]. However, the limitation of studying these elements in isolation is that we end up with multiple competing theories to explain how improvements are achieved across contexts, when it is more likely that many different change processes can be triggered *depending on the context.* These are not competing theories of change; but rather alternative theories that are contingent upon the contextual circumstances. To advance the study of context in implementation science, research must move beyond cataloguing the conditions for implementation success and integrate context within causal processes. A nuanced understanding of context cannot be disconnected from causation [25]. Therefore, propositions of conditional-causality surrounding the correlational relationship between implementation strategies and outcome improvements represent a way to bring the field closer to answering the question: what works, for whom and under what circumstances?

**Fig. 1:**
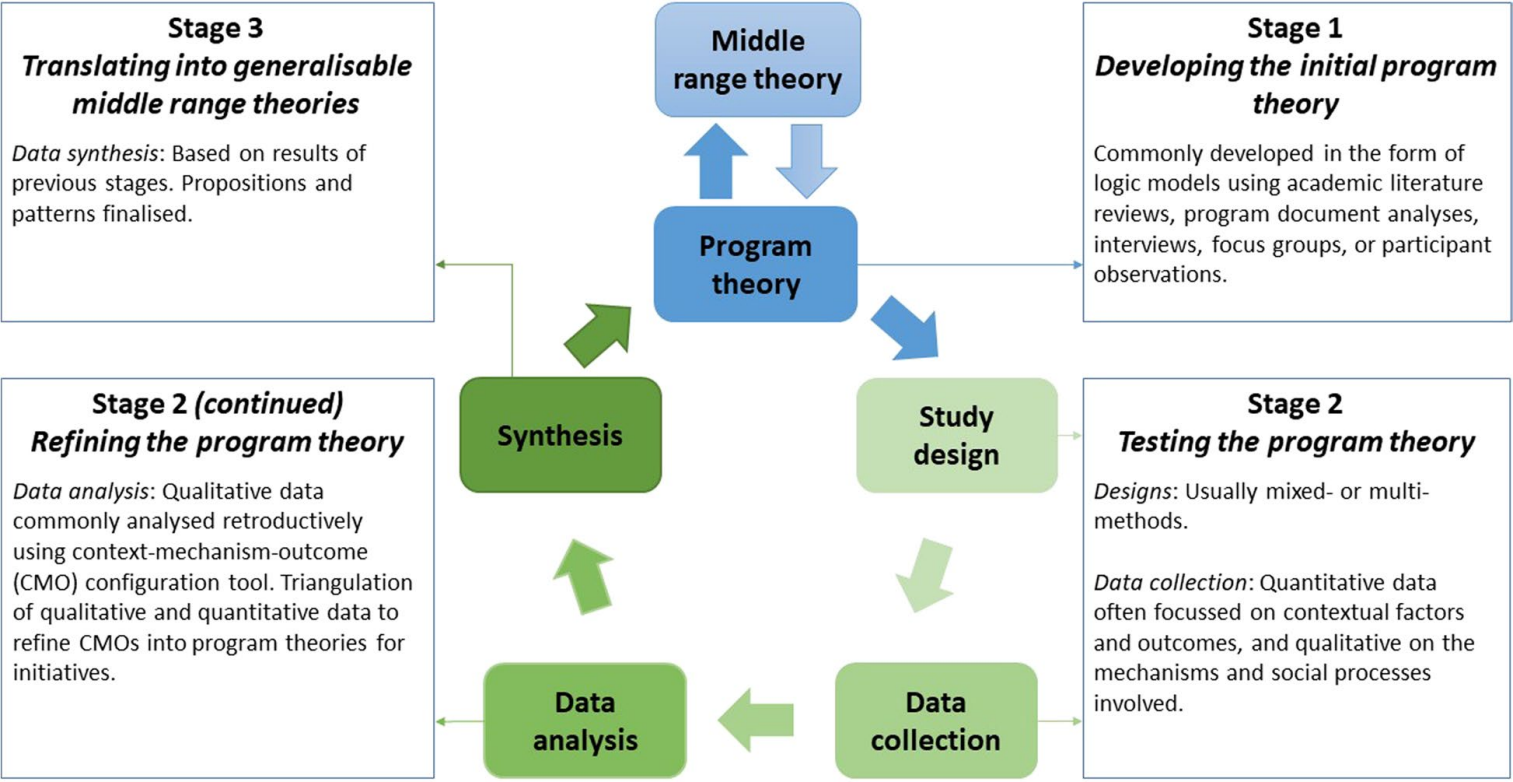
Example realist study cycle. Adapted from Pawson et al. [17], Marchal et al. [31], Mukumbang et al. [32], and Sarkies et al. [33]

The design of realist studies is method neutral, meaning multi-methods and mixed methods approaches are used [26]. Their explicit theoretical underpinnings set them apart from other theory-driven evaluations, as they consider the social responses to interventions as the primary drivers of their success rather than the interventions themselves [27, 28]. In other words, outcomes are not improved by interventions themselves but through the mechanistic responses that are triggered by those interventions. Therefore, the primary focus of inquiry centres on the “program theory”–a hypothesised description of what is supposed to be carried out in the implementation of programs and how and why that is expected to work [29, 30]. A realist study begins with the development of an initial program theory, which is then tested usually with both quantitative and qualitative data: quantitative often focussing on contextual factors and outcomes, and qualitative on the mechanisms and social processes involved. The output generated is a refined program theory, specific enough to assert testable propositions and sufficiently generalisable to apply in different situations. An example realist study cycle is illustrated in Fig. 1.

By understanding this grouping of contexts, mechanisms and outcomes, we can help clinicians bring together theory and empirical data in a way that is more representative of the actual realities they face in the health system. This framework for examining improvement initiatives uncovers the mechanistic processes or events through which they operate to enable change [17, 34]. For example, immunisation has been demonstrated empirically to reduce incidence and prevent adverse health outcomes for a range of diseases. To replicate these benefits, we need to identify *how* processes drive these improvements; in this case, the successful rollout of a vaccination program and achievement of adequate herd-immunity levels. Yet the picture still seems incomplete without uncovering why herd immunity is achieved in one setting but not another. Herd-immunity might only be achieved in certain circumstances such as societies with prosocial motives which create an imperative for people who are responsible for transmission, but do not suffer from the disease, to get vaccinated.

Note that context is considered as more than the setting in and of itself. Context encompasses the situation where improvement initiatives are implemented and crucially, the features of those situations that affect the process of improvement [35]. Articulating the circumstances under which improvement initiatives trigger the realisation of benefits in healthcare goes a long way to explaining why improvement outcomes might vary between sites. We can examine how this idea has already been leveraged to implement rapid response systems at scale. Rapid response systems are a multidisciplinary team most frequently consisting of intensive care trained personnel who are available 24 hours per day, 7 days per week for evaluation of patients who develop signs or symptoms of severe clinical deterioration [36, 37]. They are thought to work by imposing a duty of care on all health professionals to ensure early recognition and response to patients whose condition is deteriorating no matter the cause or who their treating medical doctor may be [38]. Yet, this theoretical casual mechanism is also conditional on context. The success of rapid response systems are contingent upon there being a reasonable balance between false positive and false negative response calls: too many false positives could overwhelm the workload of intensive care unit clinicians; too many false negatives could fail to achieve a timely response for many patients [38]. Illustrating that rapid response systems are only successful under these contextual circumstances allows implementers to tailor the improvement initiative to match this contextual constraint, or direct efforts to modify the context so that it is conducive to the desired changes.

### Realist implementation mechanisms and the stratification of reality

**Fig. 2:**
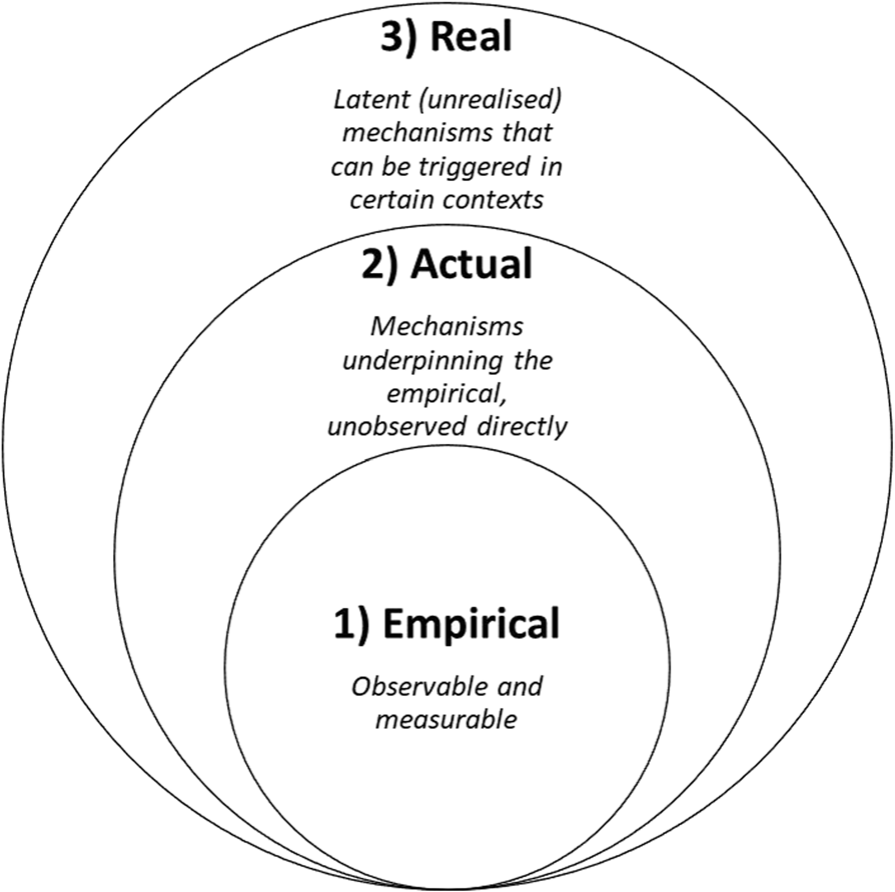
Realist concept of the ontological depth of reality stratification. Source: Adapted from Jagosh [39]

Within a realist paradigm, reality is stratified according to layers of ontological depth that make up the complete observable and non-observable world influencing the delivery of healthcare, see Fig. 2 [40]. To illustrate, according to realist philosophy, there are three “realms” that constitute reality: 1) empirical, 2) actual, and 3) real [39]. The “empirical” realm includes the observable and measurable world in which we inhabit. The “actual” refers to the underpinning mechanisms (processes and events) generating what is observed empirically but are difficult to measure or observed directly. The “real” is also unobserved directly, characterised by latent or unrealised mechanisms, which can trigger changes at the actual and empirical levels when activated under certain contexts. Returning to our earlier immunisation example, vaccines have been demonstrated at the “empirical” level to reduce incidence and prevent adverse health outcomes for a range of diseases; the “actual” level concerns the successful rollout of a vaccination program and achievement of adequate herd-immunity levels; the “real” encompasses prosocial motives that drive people who are responsible for transmission, but do not suffer from the disease, to get vaccinated.

The layering of social phenomena is what differentiates realist implementation mechanisms from other conceptualisations of causal processes. The field of implementation science, like many other disciplines, is increasingly seeking to understand the mechanisms responsible for behavioural change. Lewis et al. have advanced this concept by articulating four step causal pathway models to generate empirical evidence with clear contextual boundaries [41]. This causal pathway model assumes implementation strategies affect a single primary outcome and are sufficiently resilient to estimate effects while controlling for contextual factors. However, when dealing with complex interventions, we are faced with several interacting components and behaviours of interest across different groups and organisational levels, impacting a variety of outcomes [42].

Jolles Perez et al. addresses the challenge posed by complex health interventions by operationalising mechanisms as the “core functions” and “forms” of interventions [43]. Core functions are the steps in the change process that intervention components are seeking to facilitate [44]. Forms are the activities that may be tailored to local contexts, needed to carry out the core functions [44]. These core functions and forms of implementation strategies remain bounded within the “empirical” and “actual” layers of social processes and do not sufficiently capture the “real” latent (unrealised) mechanisms that can be triggered by certain contextual circumstances. We consider this pluralism and diversity in the conceptualisation of implementation mechanisms as a broadly positive development, as it reflects the complexity of the healthcare system and assists implementation scientists to make sense of it. We call for the coexistence of different mechanism conceptualisations in implementation science to enable their application depending on strengths and weakness in relation to the research question or practical constraints.

### Understanding change mechanisms in-situ

Recasting our focus to the mechanisms of implementation is reshaping how systems change is understood. New models are replacing the “black box” approach to evaluating improvement initiatives in healthcare, which focused on summative outcomes without sufficient explanation for how outcomes were achieved [45]. Having established the potential value of realist implementation studies, we now examine the application of this paradigm to accomplish large system change in Australian hospitals.

Healthcare systems have reorganised over the past decade to improve health outcomes valued by society [46]. Reducing unwarranted variation and maximising value for populations remains a persistent challenge large-system transformation. In New South Wales, Australia, value-based healthcare is a prominent focal point in the health reform agenda [47]. Leading Better Value Care (LBVC) is a flagship program within this reform agenda, which encompasses multiple cohort-specific improvement initiatives across over 100 health facilities. The program seeks to create shared priorities between government agencies and healthcare organisations to implement evidence-informed models of care for chronic conditions. Enabling the scaling and replication of initiative benefits across health facilities and communities is key to ensuring the success of large system healthcare transformations. A realist study of the strategies used to implement the LBVC program is being undertaken to: 1) determine how implementation strategies, contextual features and change mechanisms led to different outcomes; and 2) build models for spreading local, multiagency, evidence-informed improvement initiatives into sustainable, nationwide impacts [33].

Our realist study protocol has been published elsewhere and the findings are still under development [33]. We illustrate the foci of inquiry for two initial program theories below and demonstrate our formative realist study outputs. In brief, we conducted a three-stage mixed-methods study of the LBVC program. During Stage 1, 46 initial program theories were developed corresponding with the eight main implementation strategies deployed to support the program’s scale up. The primary information sources for this stage were academic literature reviews (~ 135 articles), program document analyses (126 documents), and key informant discussions (~ 16 stakeholders). First, relevant middle range implementation theories were identified from the extant literature to provide a conceptual framework that considered the social structure and layers of contextual circumstances (i.e., individual, interpersonal). Second, existing theoretical propositions relevant to large-system transformation in healthcare were collated and positioned within our conceptual framework of middle range implementation theories. Third, initial program theories pertaining to the LBVC program were formulated during informal discussions with key informants. Fourth, these initial program theories were modified by the project team through a dialogical approach adopted from Bohm [48]. Our full list of program theories are to be tested and refined in Stage 2 and implementation models developed in Stage 3 for reporting in future publications.

#### Example 1

**Fig. 3:**
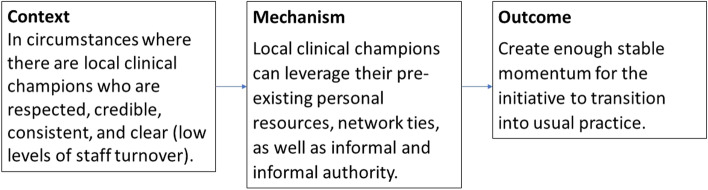
Initial Program Theory 2 for the clinical champion implementation strategy

*Clinical champions (*Fig. 3*)*: Using clinical champions to lead large-system change is often included in implementation blueprints for healthcare improvement initiatives. It is assumed to work, but such assumptions prevent an interrogation of challenges faced and exploration of the full repertoire of potential solutions. We add to the emerging conceptual models for *how* and *why* clinical champions might work [49]. Six program theories were identified which demonstrate that implementation is more successful when leaders 1) promote the visibility of the improvement initiative, 2) leverage their formal and informal influence through pre-existing and new networks, and 3) promote collective ownership. Success is constrained where there is 4) brokerage failure, 5) lack of persistence in transitioning initiatives to routine practice, 6) leadership disenfranchisement. The preliminary explanatory mechanism for Program Theory 2 is that local clinical champions who are respected, credible, consistent, and clear, can leverage their pre-existing personal resources, network ties, as well as informal and informal authority to create enough momentum for the initiative to become standard practice. We simply cannot expect champions to have the same influence in contexts with high staff turnover. This example is one of many generated from the project that goes some way to explaining how and in what circumstances clinical champions enable change.

#### Example 2

**Fig. 4:**
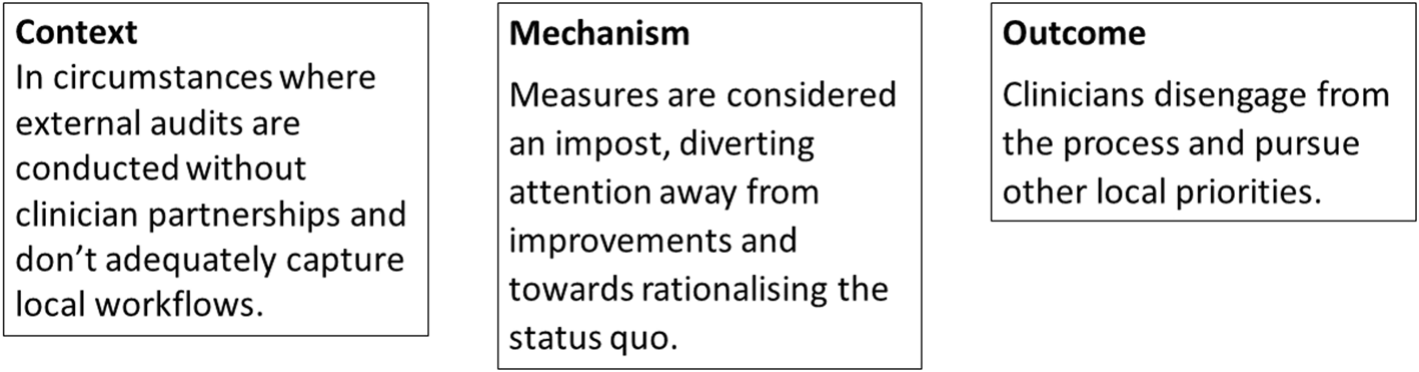
Initial Program Theory 1 for the audit and feedback implementation strategy

*Audit and feedback (*Fig. 4*)*: Audit and feedback, as mentioned earlier, is another readily applied but often misunderstood approach to implementing clinical practice change. It is thought to work through many different channels (i.e., theoretical mechanisms) [50]. Our preliminary findings highlight eight program theories that indicate the audit and feedback process used might work well when clinicians 1) feel ownership and buy in to the process, 2) can make sense of the information provided, 3) are motivated by competition, and 4) accept responsibility and accountability for changes. The success of audit and feedback was constrained by 5) rationalising of the status quo, 6) perceptions of unfairness and questions of integrity, 7) tokenistic improvements plans are developed, and 8) perceptions of threats to professional autonomy. The first program theory is explained by clinicians buying in to the process and taking ownership of the measures that represent best practice. Situations where audits are conducted by an external body without partnering with clinicians might not adequately capture local workflows. Clinicians may end up disengaged if the measures lack local meaning. Subsequently, mandatory audits conducted by an external body might be counterproductive, particularly for those clinicians whose reluctance has increased. Analysis of data from our project has generated other similar statements that describe audit and feedback driven care improvements.

In our case, we are examining a complex intervention [51]. Therefore, the implementation strategies supporting distributed leadership models through clinical champions and conducting audit and feedback cycles were not applied to one single intervention, but rather multiple interfacing initiatives for different chronic conditions: for example, establishing new outpatient ambulatory care clinics for type 2 diabetes and osteoarthritis chronic disease management, and reducing unwarranted variation in care for inpatient management of COPD, heart failure and type 1 diabetes. While realist studies examine the specific elements of a healthcare program and how they interact with local contextual circumstances, the idea is that clinical champions and audit and feedback work a certain way, to produce different outcomes, depending on contextual factors at play, that is generalisable to all large-system transformation programs. This may seem a daunting task, but one which is necessary to advance the field of implementation science.

The idea of harmonising implementation strategies, context and interventions has been eloquently articulated by Haines’ et al. to maximize usability and usefulness of implementation endeavours [52]. The key difference with a realist approach, lies in how context and mechanisms are conceptualised. Many implementation science models do not explicitly consider the mechanisms by which interventions and implementation strategies work, but instead centre on the relationship between interventions (and implementation strategies) and context, assuming their compatibility will translate into desired outcomes [7, 8, 22]. However, a realist study considers the context in relation to the mechanism: context is important in understanding theoretical propositions for how interventions work, rather than considering the compatibility between interventions and context [53]. Context is actually part of the program theory, which is the intervention, i.e., context is endogenous to the intervention, not exogenous.

These examples bring to light a nuanced understanding of conditional causal chains for implementation. It is reasonable to believe that both clinical champions and audit and feedback strategies can (and do) support the implementation of healthcare improvement initiatives at scale; but realist studies make clear that they will only work in certain circumstances. Realist work shows that positive mechanisms are only triggered if certain contextual features are in place. Applying this lens both at the outset of initiatives, but also retrospectively, provides us with the insight that an enabling and conducive context is as important as the design of interventions. If we wish to replicate healthcare improvements across different sites and settings, we need to understand these subtleties involved in the process of implementation.

## Conclusion

Exploring configurations of 1) the contextual circumstances conducive to triggering change, 2) theoretical causal mechanisms for *how* and *why* observed impacts occurred, and 3) the impacts on implementation outcomes, represents a way forward for delivering explanatory models for implementation science. A realist approach seeks to unify these three pillars influencing implementation science. Such contextually dependent implementation models enable tailoring of supportive structures, as well as articulating the expected benefits from these activities. By supporting the contemporaneous adoption and sustainment of evidence-based care, “realistic implementation” can uncover applicable evidence for and help optimise that the right care is delivered in the right setting to the right patient at the right time at the right cost.

## Data Availability

Not applicable.

## Abbreviations

ICU: Intensive Care Unit
LBVC: Leading Better Value Care
